# Epigenetic inactivation of *TCF2* in ovarian cancer and various cancer cell lines

**DOI:** 10.1038/sj.bjc.6602984

**Published:** 2006-02-14

**Authors:** K Terasawa, M Toyota, S Sagae, K Ogi, H Suzuki, T Sonoda, K Akino, R Maruyama, N Nishikawa, K Imai, Y Shinomura, T Saito, T Tokino

**Affiliations:** 1 Department of Obstetrics and Gynecology, Sapporo Medical University, Sapporo 060-8543, Japan; 2 Department of Molecular Biology, Cancer Research Institute, Sapporo Medical University, Sapporo 060-8543, Japan; 3 First Department of Internal Medicine, Sapporo Medical University, Sapporo 060-8543, Japan; 4 PRESTO, JST, Kawaguchi, Japan; 5 Department of Public Health, Sapporo 060-8543, Japan; 6 First Department of Surgery, Sapporo Medical University, Sapporo 060-8543, Japan

**Keywords:** epigenetics, methylation, ovarian cancer

## Abstract

Transcription factor 2 gene (*TCF2*) encodes hepatocyte nuclear factor 1*β* (HNF1*β*), a transcription factor associated with development and metabolism. Mutation of *TCF2* has been observed in renal cell cancer, and by screening aberrantly methylated genes, we have now identified *TCF2* as a target for epigenetic inactivation in ovarian cancer. *TCF2* was methylated in 53% of ovarian cancer cell lines and 26% of primary ovarian cancers, resulting in loss of the gene's expression. *TCF2* expression was restored by treating cells with a methyltransferase inhibitor, 5-aza-2′deoxycitidine (5-aza-dC). In addition, chromatin immunoprecipitation showed deacetylation of histone H3 in methylated cells and, when combined with 5-aza-dC, the histone deacetylase inhibitor trichostatin A synergistically induced *TCF2* expression. Epigenetic inactivation of *TCF2* was also seen in colorectal, gastric and pancreatic cell lines, suggesting general involvement of epigenetic inactivation of *TCF2* in tumorigenesis. Restoration of *TCF2* expression induced expression of *HNF4α*, a transcriptional target of HNF1*β*, indicating that epigenetic silencing of *TCF2* leads to alteration of the hepatocyte nuclear factor network in tumours. These results suggest that *TCF2* is involved in the development of ovarian cancers and may represent a useful target for their detection and treatment.

Ovarian cancer is the most deadly of gynecological malignancies, with an overall 5-year survival rate of <30%. In large part, this is because the disease usually presents at an advanced stage, as there are no overt symptoms at early stages. Ovarian cancers are morphologically and biologically heterogeneous and associated with distinct genetic alterations. For example, the serous type frequently shows p53 mutations, the mucinous type K-ras mutations, and the endometrioid type *β*-catenin mutations. The molecular pathogenesis of ovarian cancer is not fully understood, however, and it is hoped that a better understanding of the molecular mechanisms underlying the tumorigenic process will lead to earlier diagnosis, novel therapies and, ultimately, better outcomes.

DNA methylation can be a good molecular marker by which to identify genes inactivated in cancer (Jones & Baylin, 2002; Laird, 2003; Issa, 2004; Costello, 2005), and several techniques, including restriction landmark genome scanning and methylation-sensitive arbitrary primer PCR, have been used to screen for aberrantly methylated DNA fragments in tumour cells (Zardo *et al*, 2002; Yu *et al*, 2005). In addition, we have developed a novel methylation screening technique, methylated CpG island amplification (MCA), which we have used to identify DNA fragments that are hypermethylated in cancer (Toyota *et al*, 1999). And by coupling MCA to representational difference analysis (RDA) we have been able to identify those genes inactivated by hypermethylation (Toyota *et al*, 1999; Ueki *et al*, 2001; Obata *et al*, 2003; Palmisano *et al*, 2003; Yamada *et al*, 2004). When we used MCA in combination with RDA to screen for genes methylated in an ovarian cancer cell line, we observed for the first time that transcription factor 2 gene (*TCF2*) is a target for epigenetic inactivation.

*TCF1* and *TCF2*, respectively, encode the transcription factors hepatocyte nuclear factor 1*α* and *β* (HNF1*α* and HNF1*β*), two POU-homeodomain proteins that bind to an essential element in the proximal promoter sequences of albumin and many other tissue-specific genes and are associated with development, metabolism and cancer (Cereghini *et al*, 1987; Courtois *et al*, 1987; Frain *et al*, 1989; Rey-Campos *et al*, 1991). For instance, *TCF2* defects cause developmental abnormalities in multiple tissues, including kidney and pancreas (Horikawa *et al*, 1997; Haumaitre *et al*, 2005), and Bluteau *et al* (2002) identified biallelic mutations of *TCF1* in hepatic adenomas (Bluteau *et al*, 2002). Subsequently, mutations of *TCF1* also were found in colorectal cancer with MSI (Laurent-Puig *et al*, 2003) and endometrial tumours (Rebouissou *et al*, 2004), while Rebouissou *et al* (2005) also identified mutations of *TCF2* in renal carcinomas.

With that as background, our aim in the present study was to evaluate *TCF2* methylation in panels of ovarian cancer cell lines and primary ovarian cancers. Our findings indicate that *TCF2* expression is lost in ovarian cancer cell lines that show methylation and that expression is restored by treating cells with a methyltransferase inhibitor, confirming epigenetic silencing of the gene. Moreover, restoration of *TCF2* expression induced expression of *HNF4α*, a transcriptional target of HNF1*β*, indicating that epigenetic silencing of *TCF2* leads to alteration of the hepatocyte nuclear factor network in tumours. These results suggest that epigenetic inactivation of *TCF2* is involved in development of ovarian cancer.

## MATERIALS AND METHODS

### Cell lines and specimens

Eight ovarian cancer cell lines (SKOV-3, OVCAR-3, PA-1, Caov-3, TOV112D, TOV21G, SW626, OV-90), eight colorectal cancer cell lines (RKO, HCT116, DLD-1, LoVo, HT29, Colo320, SW480, Colo205), and eight gastric cancer cell lines (MKN7, MKN45, MKN74, JRST, KatoIII, AZ521, NUGC3, NUGC4), were obtained from the American Tissue Culture Collection (Manassas, VA, USA) or the JCRB (Tokyo, Japan). Seven pancreatic cancer cell lines (MIAPACA2, PK-3, PK1NC, PK-8, CF-PAC, PK-9, PK-45) were obtained from American Tissue Culture Collection or from Cell Resource Center for Biomedical Research, Institute of Development, Aging and Cancer, Tohoku University (Sendai, Japan). Seven other ovarian cancer cell lines (MH, KURA, AMOC-2, MCAS, KF, KFr, HTBOA) were described previously (Ishiwata *et al*, 1986; Kikuchi *et al*, 1986), as was the cervical cancer cell line (OMC-1) used (Ueda *et al*, 1989). All cell lines were cultured in RPMI 1640 (Life Technologies Inc., Rockville, MD, USA) supplemented with 10% fetal bovine serum and incubated under a 5% CO_2_ atmosphere at 37°C. Two cell lines that showed aberrant methylation and diminished gene expression were treated with the indicated concentration of the methyltransferase inhibitor 5-aza-2′deoxycitidine (5-aza-dC) for 96 h and/or the histone deacetylase inhibitor trichostatin A (TSA) for 24 h. The cells were then harvested and the total RNA extracted using Isogen (Nippongene, Tokyo, Japan). In addition, four samples of normal ovarian tissue and 98 ovarian cancer specimens were obtaining from Sapporo Medical University Hospital at surgery and stored at –80°C. In accordance with institutional guidelines (Ishioka *et al*, 2003), all patients gave informed consent prior to collection of the specimens.

### MCA/RDA

MCA was carried out as described previously (Toyota *et al*, 1999). We used the MCA amplicon from the OVCAR-3 ovarian cancer cell line as a tester and that from normal ovarian tissue as a driver. After two rounds of subtraction by RDA, the PCR products were digested with *Xma*I and ligated to pBluescriptII (Stratagene). The inserts were then sequenced using an ABI3100 Genetic Analyzer (Applied Biosystems, Foster City, CA, USA). Database analysis was performed using Blast and BLAT.

### Bisulphite treatment of genomic DNA

Genomic DNA was isolated from cell lines and primary tissue samples (cancerous and normal) using the phenol–chloroform method and then treated with sodium bisulphite as described previously (Clark *et al*, 1994). Briefly, 2 *μ*g of DNA were denatured for 10 min at 37°C in 2 M NaOH, after which 30 *μ*l of 10 mM hydroquinone (Sigma Chemical Co, St Louis, MO, USA) and 520 *μ*l of 3 M sodium bisulphite were added. The mixture was then incubated for 16 h at 50°C, and the modified DNA was purified using a Wizard DNA Purification System (Promega, Madison, WI, USA). After treating the DNA with NaOH a second time, the resultant DNA precipitate was resuspended in 20 *μ*l of TE buffer and stored at −80°C until use.

### Combined bisulphite restriction analysis

The methylation status of *TCF2* was examined using combined bisulphite restriction analysis (COBRA), a semiquantitative bisulphite-PCR analysis (Xiong & Laird, 1997). Primers were designed so that both methylated and unmethylated DNA would be amplified equally. PCR was carried out in a 50-*μ*l volume containing 1 × PCR buffer (67 mM Tris-HCl (pH 8.8), 16.6 mM (NH_4_)_2_SO_4_, 6.7 mM MgCl_2_, and 10 mM *β*-mercaptoethanol), 0.25 mM dNTP mixture, 0.5 *μ*M each primer and 1.0 U of Hot Start Ex-Taq polymerase (TaKaRa). The oligonucleotide sequences used for bisulphite-PCR were 5′-GGGGTYGAGTTYGATATTAAGT-3′ (TCF2COBRA-F) and 5′-TACCTAAACATCCRATCCACCT-3′ (TCF2COBRA-R). The PCR products were digested with the restriction enzyme *Tai*I (Fermentas Life Sciences, Ontario, Canada), which cleaves CpG sites retained because of methylation. After ethanol precipitation, the DNA was subjected to 2.5% agarose gel electrophoresis and stained with ethidium bromide.

### Direct-sequencing of Bisulphite-PCR products

The PCR products obtained with COBRA were used for direct-sequencing. Briefly, 10 *μ*l of bisulphite-PCR product were electrophoresed in 1% SeaPlaque agarose gel, excised, purified using a PCR Purification System (Promega, Madison, WI, USA) and eluted in 50 *μ*l of water. Samples (5 *μ*l) were then used as templates for the sequencing reaction, which was carried out using a BigDye terminator cycle sequencing kit (Applied Biosystems, Foster City, CA, USA) with an ABI PRISM 3100 sequencer according to the manufacturer's guidelines (Applied Biosystems).

### Reverse transcription–polymerase chain reaction

Expression of *TCF2* and *HNF4α* was analyzed using reverse transcription–polymerase chain reaction (RT—PCR). Total RNA was extracted from cell lines using Trizol (Life Technologies Inc., Gaithersburg, MD, USA) according to the manufacturer's instructions. The RT reaction was carried out with 2 *μ*g of total RNA using a SuperScript II First-Strand Synthesis System (Invitrogen Inc., Grand Island, NY, USA) with random primer. PCR was carried out in solution containing 1 × PCR buffer (TaKaRa), 200 *μ*M each deoxynucleotide triphosphate, 2.5 pmol of each primer, 1 U of ExTaq polymerase (TaKaRa) and 5% (v/v) DMSO. The oligonucleotide primer sequences used were 5′-CTATGACACACCTCCCATCCTCAAG-3′ (TCF2RT-F), 5′-GTCTGGTTGAATTGTCGGAGGATCT-3′ (TCF2RT-R), 5′-CGGCAGTGCGTGGTGGACAAAGAC-3′ (HNF4-*α*RT-F), and 5′-CACACACATCTGCGATGCTGGCAAT-3′ (HNF-4*α*RT-R). The PCR cycling protocol consisted of 1 cycle at 95°C for 5 min and 32 cycles at 95°C for 30 s, 60°C for 30 s and 72°C for 45 s. The housekeeping gene *GAPDH* served as an internal control to confirm the success of the RT reaction. The PCR products were subjected to 2.5% agarose gel electrophoresis.

For quantitative analysis, real-time PCR was carried out using a TaqMan PCR system (Applied Biosystems). PCR was carried out in 50 *μ*l of solution containing 1 *μ*l of cDNA, 2.5 pmol of each primer and 25 *μ*l of TaqMan PCR mixture (Applied Biosystems). The PCR cycling protocol consisted of 1 cycle at 95°C for 5 min and 40 cycles at 95°C for 30 s and 60°C for 1 min. Fluorescent signals were detected using an ABI 7000 Prism 7000 (Applied Biosystems), and the accumulation of PCR product was measured in real time as the increase in the fluorescent signal. Data were analysed using ABI Prism 7000 SDS Software (Applied Biosystems). The probes and primer sets were ABI Assay on Demand and Ref Hs00172123 for *TCF2* and Hs00230853 for *HNF4α* (Applied Biosystems).

### Mutational analysis

In all, 10 pairs of primers for individually amplifying and screening exons 1–9 of *TCF2* were used for amplifying genomic DNA. Spanning the entire coding region of the gene, these primers were specified by a Human BLAT Search (Chr17q12, position: 33,120,547–33,179,182), and their sequences are available upon request. PCR was carried out in a 50-*μ*l reaction mixture containing 1 × PCR buffer (TaKaRa), 1 *μ*M primers, 0.25 mM dNTP mixture and 1.0 U of Hot Start Taq polymerase (TaKaRa). The amplified PCR products were electrophoresed in 1% Seaplaque gels, excised, purified using a Gel purification system (Qiagen, Hilden, Germany) and sequenced. The primer sequences and PCR conditions used for mutational analysis are available upon request.

### Chromatin immunoprecipitation and PCR

Chromatin immunoprecipitation was carried out as described previously (Ueno *et al*, 2004). Briefly, cells were incubated in 1% formaldehyde for 10 min at 37°C. The nuclei were then collected, sonicated to yield fragments ranging in size from 300 to 2000 bp, and immunoprecipitated with anti-histone H3 antibody (Upstate Biotechnology, Lake Placid, NY, USA), which specifically recognizes the diacetylated lysine residues (lysines 9 and 14) of histone H3. The chromatin was recovered using protein A Sepharose. Real-time PCR was then performed using 1 *μ*l of chromatin DNA as shown above. The primer sequences used were 5′-TGGATTTGGGGTTTGCTTGTGA-3′ (TCF2ChIP-F) and 5′-GACGTGAGCTTGGACACCATTTTC-3′ (TCF2ChIP-R). Standard curves relating initial template copy number to fluorescence and amplification cycle were generated using the amplified PCR product as a template, and were used to calculate the DNA copy number in each sample. Ratios of the intensities of the *TCF2* and *GAPDH* signals were used as a relative measure of the level of *TCF2* expression in each specimen.

### Statistical analysis

The statistical analysis was carried out using StatView software (SAS Institute Inc., Cary, NC, USA). Fisher's exact test (two-sided) was used to determine the association between *TCF2* methylation and clinicopathological features. Values of *P*<0.05 were considered significant. Overall survival time was defined as the period between the diagnosis of ovarian cancer and the time of death. Differences between survival curves were assessed by Kaplan–Meier analysis using the log-rank test.

## RESULTS

### Identification of *TCF2* as a target for epigenetic inactivation in ovarian cancer

**Figure 1:**
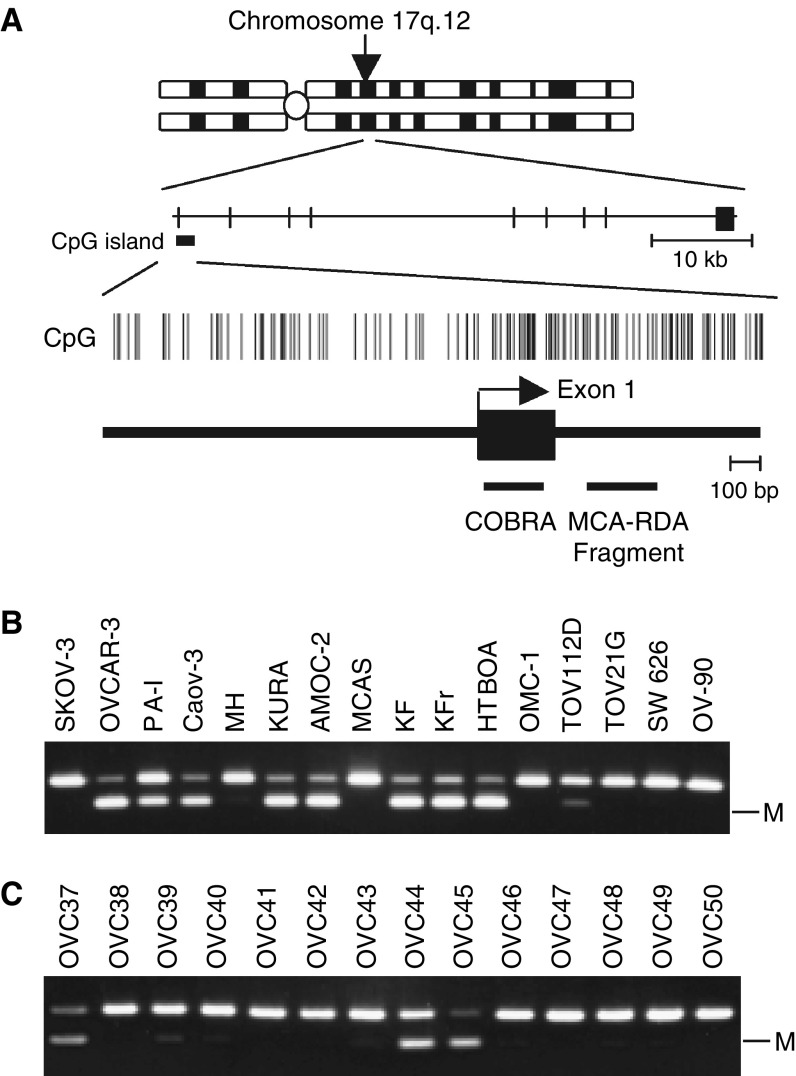
Identification of *TCF2* as a target for methylation in ovarian cancers. (**A**) Schematic diagram of the CpG island of *TCF2*. The chromosomal region in which *TCF2* is situated is shown at the top. Vertical bars indicate CpG sites. Exon is shown by a solid box with an arrow indicating the transcription start site. The region analysed using COBRA and the position of the clone obtained with MCA are shown by respective horizontal bars. (**B**) Analysis of *TCF2* methylation in ovarian cancer cell lines using COBRA. PCR products contain a restriction site for *Tai*I, which recognizes ACGT. The PCR products are digested only when the CpG sites were retained after bisulphite treatment because of methylation. The cell lines tested are shown on the top. M: methylated alleles. (**C**) Analysis of *TCF2* methylation in primary ovarian cancer. The numbers of the cases are shown above.

Using MCA coupled with RDA, we recovered DNA fragments located within intron 1 of *TCF2* (Figure 1A). The 5′ region of *TCF2* contains a CpG rich region that fulfills the criteria for a CpG island (observed CpG: expected CpG=0.821, GC content 58.9%, length 3.4 kb). Then when we used COBRA to examine the methylation status of this region in 15 ovarian and one cervical cancer cell line (Figure 1B), we found it to be densely methylated in eight (53%) of the ovarian cancer cell lines, but not in the cervical cancer cell line. Moreover, using the same methodology, we determined that *TCF2* was aberrantly methylated in 26 of 98 (26.5%) primary ovarian cancers, but not in any of the four samples of normal ovarian tissue analysed (Figure 1C). Apparently, methylation of *TCF2* is a cancer-specific event and is not a cell line-specific one.

**Figure 2:**
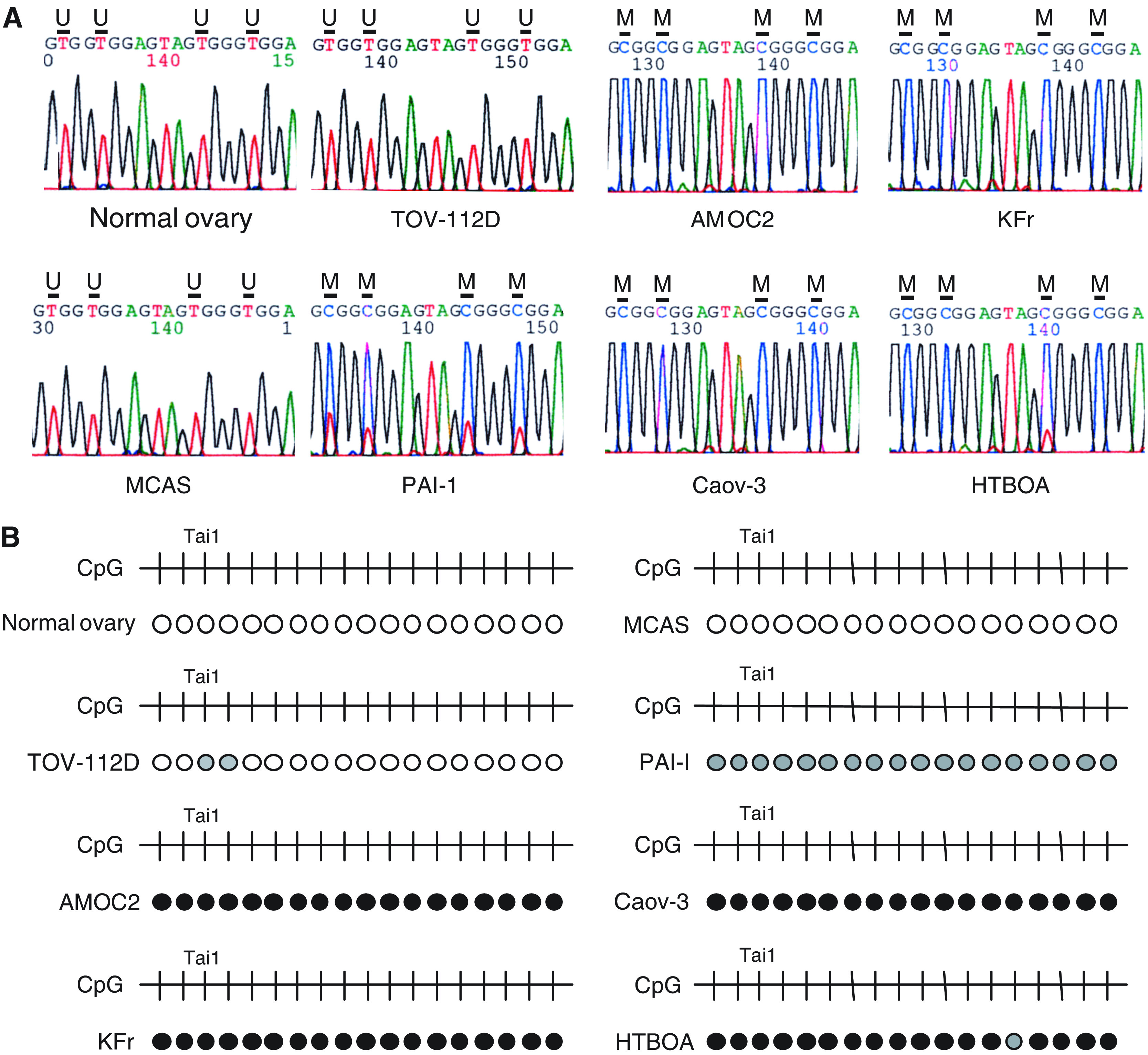
Bisulphite-sequencing analysis of *TCF2* in ovarian cancer cell lines. (**A**) PCR products used for COBRA analysis were electrophoresed in agarose, excised, purified and directly sequenced. In normal ovary and TOV112D and MCAS cells, all of the cytosines within the CpG sites were substituted with thymines. In AMOC2, KFr, Caov-3 and HTBOA cells, all cytosines in the CpG sites remained cytosines. In PAI-1 cells, the CpG sites contained a mixture of cytosines and thymines, as methylation was partial. U: unmethylated CpG sites; M: methylated CpG sites. (**B**) Summary of bisulphite-sequencing. Unmethylated CpG sites are indicated by open circles, and methylated CpG sites are indicated by filled circles. CpG sites that show a mixed pattern of methylated and unmethylated alleles are shown in the grey circles. Cell lines are indicated on the left. The CpG sites in the region analysed are indicated by vertical bars (top). The CpG site examined by COBRA analysis is indicated as *Tai*I.

**Figure 3:**
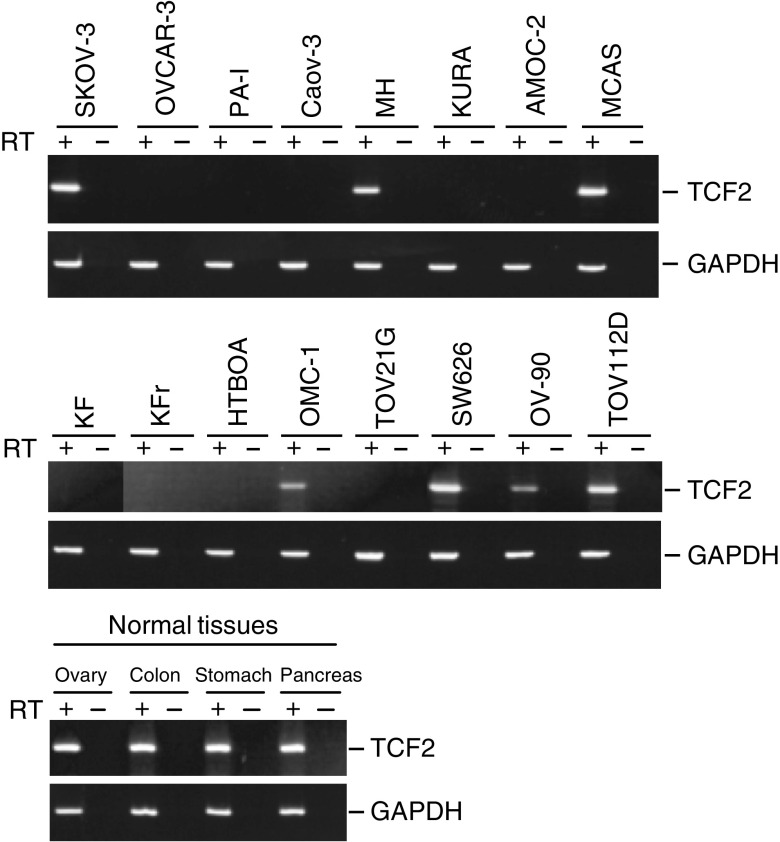
RT–PCR analysis of TCF2 mRNA expression in ovarian cancer cell lines. Controls consist of carrying out PCR reactions without reverse transcription (−). The cell lines examined are shown above the gels. Expression of GAPDH was evaluated to confirm the integrity of cDNA.

To confirm whether the methylation detected by COBRA reflects the overall level of methylation in the 5′ region of *TCF2*, we carried out bisulphite-sequencing of the gene (Figure 2A and B). Again, methylation of *TCF2* was not detected in normal ovarian tissues. On the other hand, the ovarian cancer cell lines shown by COBRA to be methylated also showed dense methylation over all their CpG islands, while those shown by COBRA not to be methylated showed no methylation of their CpG islands (Figure 2A and B). To determine whether methylation of *TCF2* is correlated with gene silencing, we used RT–PCR to evaluate the gene's expression in 15 ovarian and one cervical cancer cell lines (Figure 3). No expression of *TCF2* was detected in any of the nine cell lines that showed methylation; that is, methylation was significantly associated with an absence of TCF2 mRNA (*P*<0.001, Fisher's exact test, two-sided). By contrast, all seven cell lines that showed no methylation expressed the transcript, indicating a role for DNA methylation in the silencing of *TCF2*.

To determine whether *TCF2* is inactivated by mutation, we first carried out direct sequence analysis of *TCF2* using DNA from ovarian cancers. Using 10 sets of primers that respectively amplified exons 1–9, we examined 15 cell lines and 33 primary ovarian tumours, and discovered two base substitutions in the introns, which were determined to be polymorphisms (data not shown, and Supplementary Figure 1). Thus, inactivation of *TCF2* appears to be caused by epigenetic changes rather than genetic ones.

### Silencing of *TCF2* in human tumour cell lines

**Figure 4:**
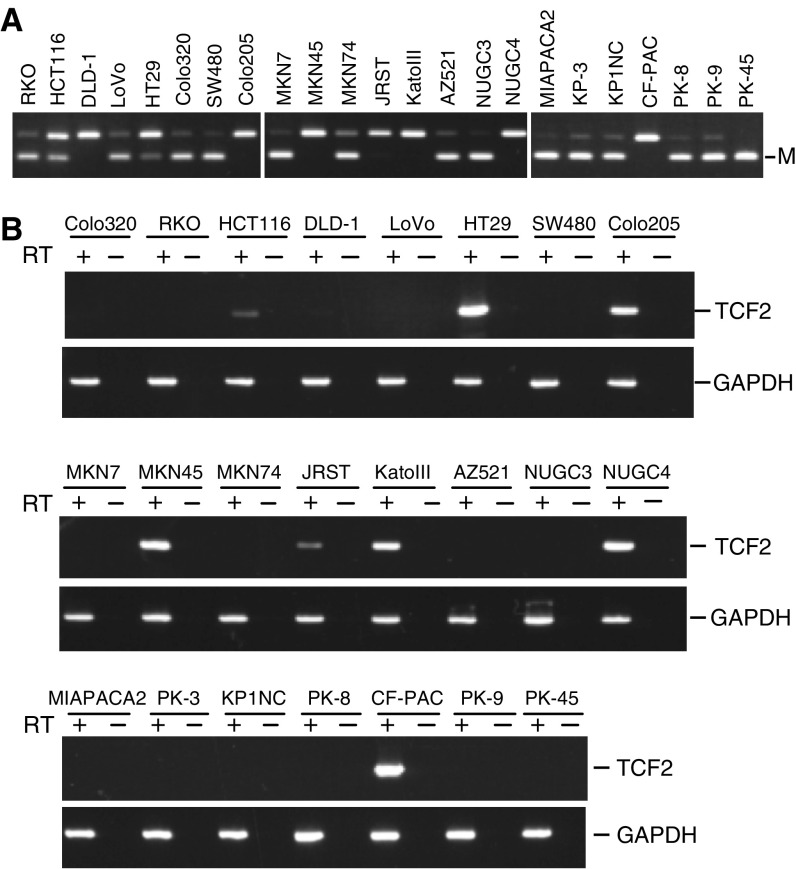
Epigenetic silencing of TCF2 mRNA expression in human tumour cell lines. (**A**) Analysis of *TCF2* methylation using COBRA in colorectal, gastric and pancreatic cancer cell lines. Cell lines are shown on the top. (**B**) RT–PCR analysis of TCF2 mRNA expression.

We next carried out COBRA using DNA from a panel of human cancer cell lines to determine the frequency with which *TCF2* was methylated in other tumour types (Figure 4A). Methylation of *TCF2* was detected in six of eight (75%) colorectal, four of eight (50%) gastric and six of seven (85.7%) pancreatic cancer cell lines (Figure 4B). Moreover, as in ovarian cancer, expression of *TCF2* was absent in cell lines that showed methylation. One colorectal cancer cell line, DLD-1, also did not express *TCF2*, although this cell line did not show methylation.

### The role of DNA methylation and histone deacetylation in the silencing of *TCF2*

**Figure 5:**
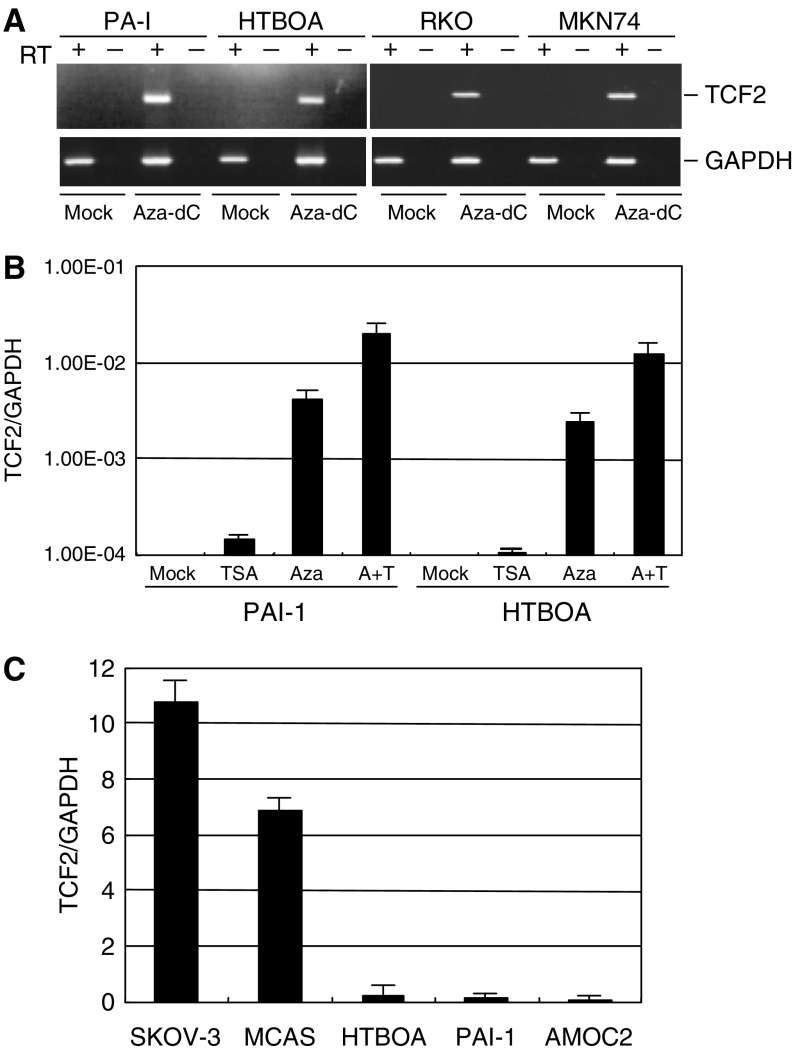
Silencing of *TCF2* is associated with DNA methylation and histone deacetylation. (**A**) Restoration of TCF2 mRNA expression by 5-aza-dC. RT–PCR showed reactivation of TCF2 mRNA expression in two ovarian and two gastrointestinal cancer cell lines following treatment with 2 *μ*M of 5-aza-dC for 72 h. (**B**) Synergistic effect of 5-aza-dC and TSA. PAI-I and HTBOA ovarian cancer cells were treated with 5-aza-dC and/or TSA, after which TaqMan PCR was carried out. (**C**) Acetylation status of histone H3 in cell lines with and without TCF2 methylation. Acetylation of histone H3 was assessed using ChIP assays followed by real-time PCR. In both (**B**, **C**) levels of TCF2 mRNA are normalised to those of GAPDH mRNA.

The role of DNA methylation in the silencing of *TCF2* was further confirmed by our finding that treating cells with the methyltransferase inhibitor 5-aza-dC (1 *μ*M) restored gene expression. Of four methylated cell lines tested, all showed restored *TCF2* expression following treatment with 5-aza-dC (Figure 5A). By contrast, expression of *TCF2* was not restored by the histone deacetylase inhibitor TSA, but when combined with a low dose of 5-aza-dC, TSA exerted a synergistic effect augmenting gene expression (Figure 5B). Using ChIP assays to examine the acetylation status of histone H3, we found that DNA methylation was associated with a low level of histone acetylation, indicating that histone deacetylation, too, plays a role in the silencing of *TCF2* (Figure 5C).

### The role of *TCF2* methylation in the regulation of HNF4*α*

**Figure 6:**
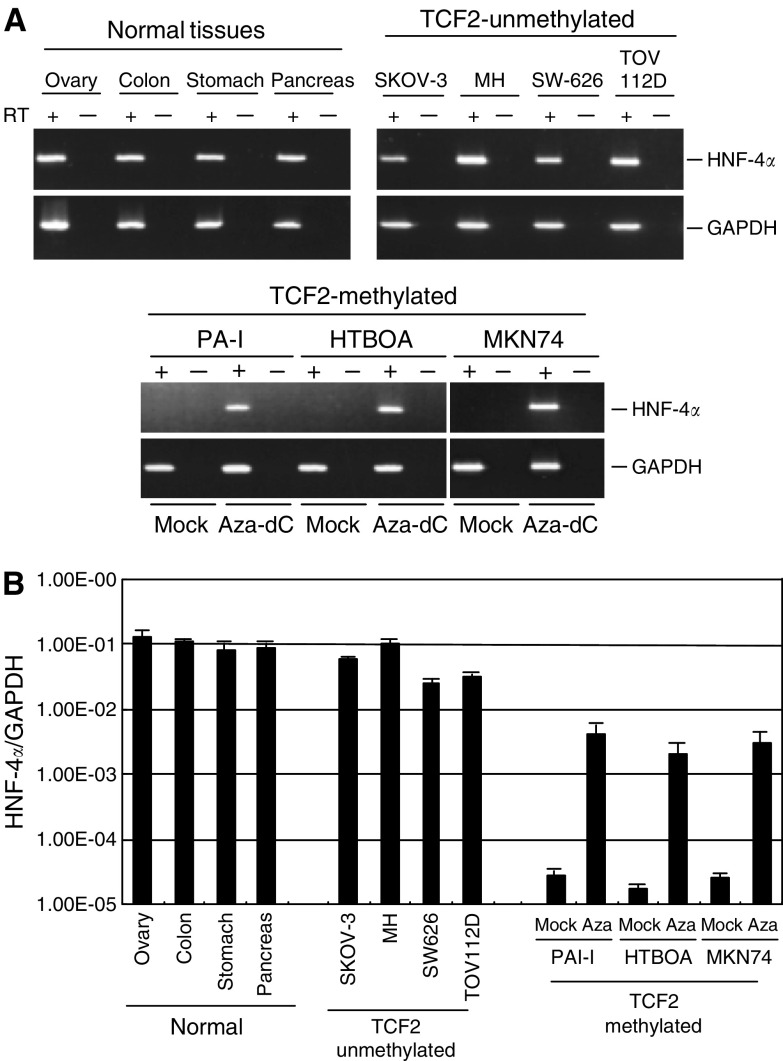
Analysis of HNF4α mRNA expression. (**A**) Expression of HNF-4*α* in cancer cell lines and normal tissues was analysed by RT–PCR. Cell lines, *TCF2* methylation statuses, and tissue types are shown on the top. PAI-1, HTBOA and MKN74 cells were treated with 5-aza-dC (Aza-dC) and examined for restoration of HNF-4*α*. (**B**) Quantitative analysis of HNF-4*α* mRNA expression using real-time PCR. Levels of HNF-4*α* mRNA were normalised to those of GAPDH mRNA.

We next evaluated the expression of *HNF4α*, one of the transcriptional targets of HNF1β, in cell lines with and without *TCF2* methylation (Figure 6A). Expression of *HNF4α* was detected in all cancer cell lines that expressed *TCF2*. By contrast, cell lines that showed *TCF2* methylation showed little or no *HNF4α* expression. Notably, treating cells with 5-aza-dC upregulated expression of *HNF4α* (Figure 6B and C). As the 5′ region of *HNF4α* does not contain a CpG island, the effect of 5-aza-dC on the gene's expression is likely not associated with demethylation of its promoter, but instead underscores the importance of *TCF2* expression, and thus HNF1*β*, in the regulation of HNF4*α*.

### Clinicopathological features of primary ovarian cancer with or without *TCF2* methylation

Finally, we examined the clinicopathological features of ovarian cancers with and without *TCF2* methylation (Table 1). Aberrant methylation of *TCF2* was detected in 12 of 29 (41.3%) serous tumours, three of 12 (25.0%) mucinous tumours, eight of 28 (28.6%) endometrioid tumours and three of 12 (25.0%) others (undifferentiated, mixed type), but in none of the 17 (0%) clear cell tumours. Methylation of *TCF2* was detected significantly less frequently in the clear cell phenotype than the other types (0% vs 32.1%, Fisher's Exact test, *P*=0.005), but there was no significant association with any other clinicopathological feature (patients' age, FIGO clinical stage, and pathological grade). There was also no correlation between the patients' prognoses and the *TCF2* methylation status (data not shown, and Supplementary Figure 2).

## DISCUSSION

The transduction network associated with tumorigenesis and the progression of ovarian cancer is not fully understood, although numerous studies indicate that DNA methylation plays a key role in human neoplasia (Jones & Baylin, 2002; Laird, 2003; Issa, 2004; Costello, 2005). Although methylation of several cancer-related genes has been reported in ovarian cancer (Yu *et al*, 1999; Terasawa *et al*, 2004), the methylated genes have not yet been fully characterised. Using the MCA genome screening technique coupled with RDA, we identified *TCF2* as a novel target of epigenetic inactivation through methylation in ovarian cancer. The Methylation of *TCF2* was cancer-specific and, together with histone deacetylation, may be involved in tumorigenesis in a subset of ovarian cancers. It has been reported that patients with germ line mutation of *TCF2* develop renal tumours (Bellanne-Chantelot *et al*, 2004), and *TCF2* mutations have been identified in renal cell cancer (Rebouissou *et al*, 2005). On the other hand, no *TCF2* mutation has been previously observed in ovarian cancer (Rebouissou *et al*, 2005), which is consistent with our present finding that *TCF2* is not mutated in ovarian cancer. Apparently, *TCF2* expression is silenced by an epigenetic rather than genetic mechanism. That methylation of *TCF2* was also detected in colorectal, gastric and pancreatic cancer cell lines suggests *TCF2* is prone to methylation in many types of cancer and may thus play a general role in tumorigenesis. In addition, one ovarian cancer cell line, TOV112D, and one colorectal cancer cell line, DLD-1, did not express TCF2, although little or no methylation was detected in tumours. Genes silenced in cancer are often not expressed in cancer cell lines (Suzuki *et al*, 2002), and their silencing may be caused by mechanisms other than methylation, for example by the absence of a transcription factor or histone modification (Kondo *et al*, 2004).

The methylation profile of ovarian cancer may be a useful diagnostic marker with which to predict recurrence, resistance to chemotherapy and survival (Balch *et al*, 2004). We and others have reported that TMS1, 14-3-3sigma and WT1 genes are methylated more frequently in clear cell tumours than in other tumour types (Kaneuchi *et al*, 2004; Terasawa *et al*, 2004; Kaneuchi *et al*, 2005), whereas methylation of SFRP1, 18S and 28S ribosomal DNA genes is more frequently detected in nonclear cell tumours, such as the serous type (Takada *et al*, 2004; Chan *et al*, 2005). Although the molecular mechanisms responsible for the differing methylation profiles in different tumour types remains unclear, gene methylation may have relevance in the clinical situation. For instance, clear cell ovarian cancers are reportedly more resistant to chemotherapy than other types (Sugiyama *et al*, 2000), and this difference in sensitivity may be caused in part by differences in the genes methylated in clear cell and nonclear cell tumours. In fact, Teodoridis *et al* (2005) reported that methylation of genes involved in DNA repair was significantly associated with response to chemotherapy, while Chan *et al* (2005) reported that methylation of 18S and 28S ribosomal DNA genes is associated with longer progression-free survival in ovarian cancer. Taken together, these results suggest that differences in the epigenetic signatures between clear cell and other types of ovarian cancer may be a useful diagnostic indicator.

The specific role of *TCF2* methylation in the development and progression of ovarian cancer remains unknown, but *TCF2* mutations are known to affect expression of downstream genes such as *HNF4α*, *PKHD1* and *UMOD* (Tanaka *et al*, 2004; Rebouissou *et al*, 2005). We showed expression of *HNF4α* to be well correlated with that of *TCF2* and to be upregulated by inducing *TCF2* expression using a methyltransferase inhibitor, which suggests alterations in the hepatocyte nuclear factor network can be reversed by inducing *TCF2* through demethylation of the gene. Consistent with that idea, Lazarevich *et al* (2004) reported that expression of *HNF4α* is downregulated in hepatocellular carcinoma and its restoration suppresses tumour growth. It has also been reported that HNF4*α* is downregulated in other types of tumours (Lemm *et al*, 1999) and that it induces expression of endothelial Fas ligand (FasL), which prevents cancer cell transmigration (Osanai *et al*, 2002). Collectively then, the summarised findings suggest that impairment of the HNF1*β*/HNF4*α* signalling network may lead to tumour formation. Similar epigenetic inactivation of a critical regulator of a transcriptional network has been reported for several other genes, including *estrogen receptor* (Leu *et al*, 2004) and *GATA* (Akiyama *et al*, 2003). Epigenetic inactivation of such transcription factors may affect multiple downstream genes, causing aberrant alteration of signalling pathways involved in a wide array of cellular functions, including growth, differentiation and apoptosis. Conversely, restoration of such transcription factors may lead to upregulation of genes throughout the entire signalling network and restoration of proper function. That said, further study will be necessary to definitively determine the role of epigenetic inactivation of *TCF2* in the molecular mechanism of tumorigenesis and progression of ovarian cancer and its utility as a therapeutic target.

## Figures and Tables

**Table 1:** Clinicopathological features of ovarian cancer with and without methylation of *TCF2*

	**Methylated (%)**	**Unmethylated (%)**
Stage^a^		
I	8 (25.8)	23 (74.2)
II	1 (33.3)	2 (66.7)
III	15 (26.3)	42 (73.7)
IV	2 (25)	5 (75)
		
Histology^b^		
Serous	12 (41.4)	17 (58.6)
Mucinous	3 (25.0)	9 (75.0)
Endometrioid	8 (28.6)	20 (71.4)
Clear cell	0 (0)	17 (100)
Others	3 (25.0)	9 (75.0)

a Methylation of *TCF2* was not associated with the stage of the tumours.

b Methylation of *TCF2* was less frequently observed in clear cell types than other types (*P*=0.005, Fisher's exact test, two-sided).
