# Supplementary material for: Epigenetic inactivation of TCF2 in ovarian cancer and various cancer cell lines
Source: Br J Cancer. 2006 Feb 14;94(6):914–21. doi: 10.1038/sj.bjc.6602984 (PMC2361363; doi:10.1038/sj.bjc.6602984)
Supplement: Supplementary Information [file 94-6602984x1.pdf]

Supplementary information for reviewers

Figure 1

Primers for mutation analysis of TCF2			
	Sequences	Annealing temprature (°C)	Sizes of PCR products (bp)
Exon 1A	F: 5'-GGTGGAGGGGTTCCTGGATTTG-3'	55	310
	R: 5'-GGCCGTTGGTGAGAGTATGGAAG-3'		
Exon 1B	F: 5'-GGCCTTGGAGGAGTTGCTGCCATC-3'	60	307
	R: 5'-GCTCCAGGGGTTCGGGTGGGTC-3'		
Exon 2	F: 5'-GGTAGCACCCCCTAGAAAAAGAATGT-3'	60	327
	R: 5'-GGGCAAAGGTCACTTCAGGTTGAG-3'		
Exon 3	F: 5'-GTCTGTCTGCTGAGTGAAGGCTA-3'	60	379
	R: 5'-GGTTCCTGGGTCTGTGTACTTG-3'		
Exon 4	F: 5'-CCAACCAAGACTGCTGTGATTGTGTG-3'	60	370
	R: 5'-GTTGGGTTGCCGAGGCAGTG-3'		
Exon 5	F: 5'-GAGGTGCCGAGTCATTGTTCCAG-3'	60	312
	R: 5'-GGCAGGCCTTGTGAGAAGTTGTG-3'		
Exon 6	F: 5'-ACATCGTGTTGGAAACTGCTC-3'	55	285
	R: 5'-CAGTTATTTTTCACCACATTAATTTG-3'		
Exon 7	F: 5'-CCTCTCCTTATCCCAGGAGCTGT-3'	60	300
	R: 5'-GTTGGCCACTGAGGGTCCTGA-3'		
Exon 8	F: 5'-CCACTATAACCACCAGCCCTTTTATC-3'	60	312
	R: 5'-CTCTGCACATCCATGGCCTTATC-3'		
Exon 9	F: 5'-CTCTTTGCTGGTTGAGTTGGGCATC-3'	60	230
	R: 5'-CCGGTCAGGTCACTGGGCTTTTC-3'		

Polymorphism in intron 7

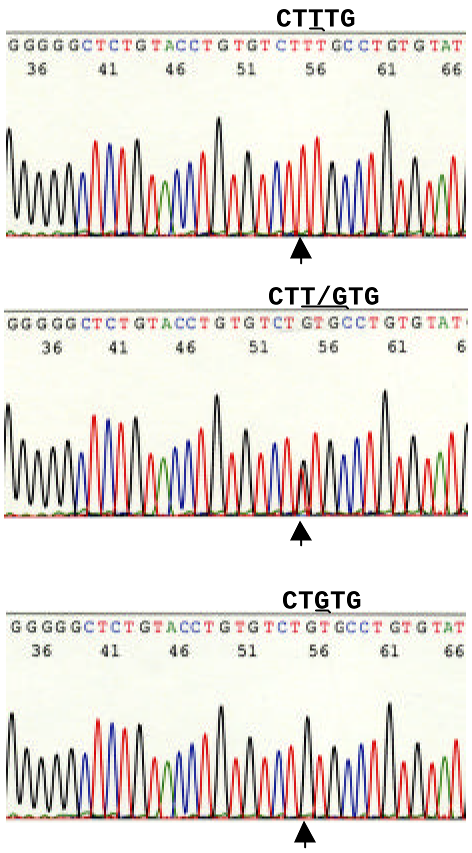

Figure 2

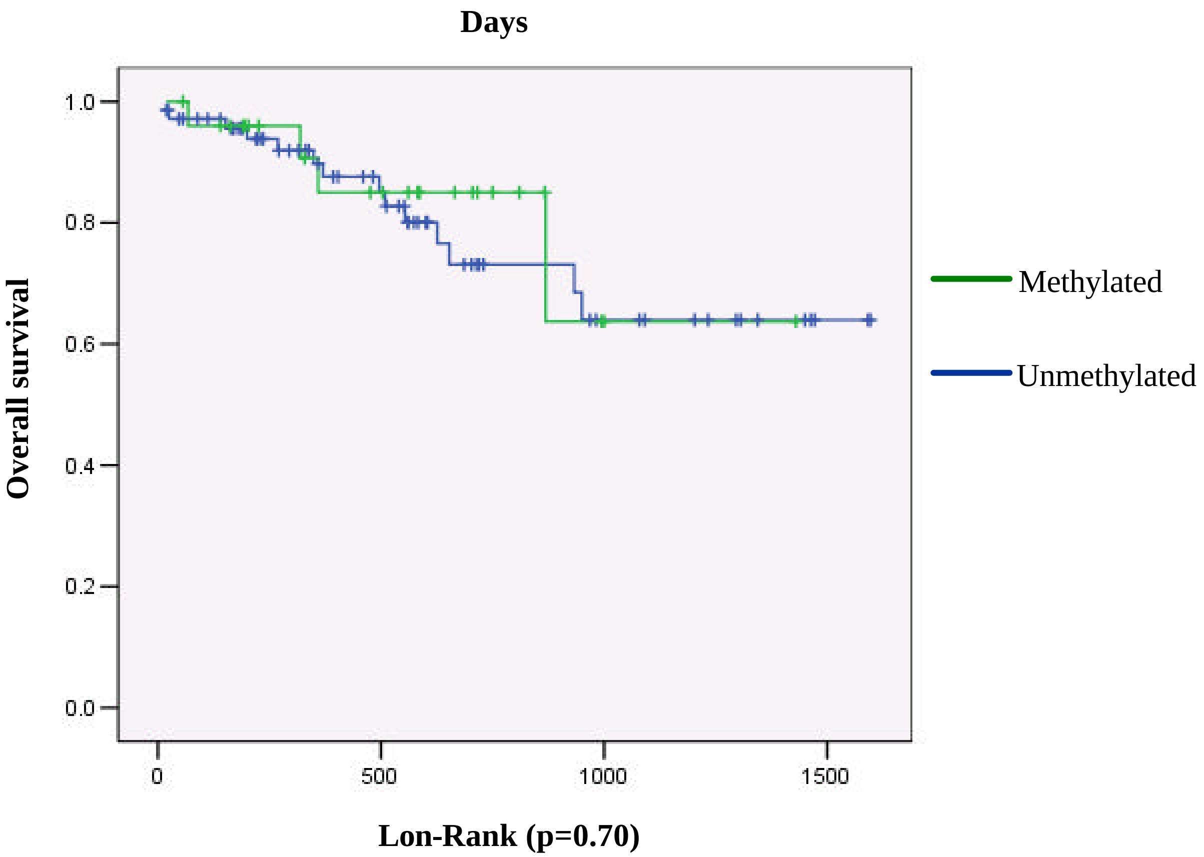

Overall survival in 98 ovarian cancers with or without TCF2 methylation.
